# Reproductive phases coincide with changes in morphology and photosynthetic physiology in an endangered cycad species

**DOI:** 10.1093/conphys/coad020

**Published:** 2023-04-25

**Authors:** Christopher P Krieg, Sophia Gosetti, James E Watkins Jr, M Patrick Griffith, Katherine A McCulloh

**Affiliations:** Department of Botany, University of Wisconsin, 340 Lincoln Dr., Madison, WI 53706, USA; Department of Botany, University of Wisconsin, 340 Lincoln Dr., Madison, WI 53706, USA; Glacial Lakes Conservancy, 529 Ontario Ave, Sheboygan, WI 53081, USA; Department of Biology, Colgate University, 13 Oak Dr., Hamilton, NY 13346, USA; Montgomery Botanical Center, 11901 Old Cutler Rd., Coral Gables, FL 33156, USA; Department of Botany, University of Wisconsin, 340 Lincoln Dr., Madison, WI 53706, USA

**Keywords:** sterile, reproduction, photosynthesis, functional traits, fertile, cycads, conservation

## Abstract

Cycadales is highly endangered and one of the oldest dioecious gymnosperm lineages, making their reproductive biology highly relevant to conservation efforts and our understanding of the impact of dioecy, yet cycad reproductive ecophysiology is poorly understood. We examined how the costs associated with reproduction may impact basic physiological variation in cycad species. Specifically, we measured traits related to functional morphology and photosynthetic physiology in sterile and fertile staminate plants (‘males’) of *Zamia portoricensis*. Light response curves showed that sterile plants had greater light-use efficiency and maximum photosynthetic capacity per area compared with fertile plants. However, fertile and sterile plants exhibited similar respiration rates. We found significantly more nitrogen in leaves of fertile individuals, but similar nitrogen isotope composition and no differences in carbon content between sterile and fertile individuals. Despite having lower leaf-level photosynthetic rates, fertile plants had greater canopy-level photosynthesis than sterile plants, which was achieved by increasing leaf number and total leaf area. Our data suggest that sterile individuals may have greater light demands relative to fertile individuals, and fertile individuals may have greater nitrogen demands, which may be critical for successful reproductive events in staminate plants of the endangered cycad, *Z. portoricensis*.

## Teaser

Cycadales is nominally the most endangered plant group on Earth, yet so much of their basic biology remains unknown which limits conservation strategies. We examined how key aspects of plant function may change with reproductive status in a tropical endangered cycad. We found that sterile individuals may have greater light demands relative to fertile individuals, and fertile individuals may have greater nitrogen demands, which may be critical for successful reproductive events in this endangered cycad.

## Introduction

Cycads first appeared around 300 million years ago, making the lineage one of the oldest extant seed-bearing plant groups (Nixon *et al.,* 1994; Taylor *et al.,* 2009). Today, Cycadales is considered the most endangered plant group on Earth, with nearly 70% of species threatened with extinction (Donaldson, 2003; The IUCN Red List of Threatened Species, 2010). This ancient plant group is threatened by many anthropogenic factors, including habitat destruction, illegal poaching and climate change (Donaldson, 2003). Conservation agencies and practitioners around the world have been working toward restoring and protecting many cycad species from extinction through effective preservation of genetic variation (Griffith *et al.,* 2015), habitat restoration and identification of areas suitable for reintroductions (Swart *et al.,* 2018) and characterization of growth and reproductive biology (Negrón-Ortiz *et al.,* 1996; Krieg *et al.,* 2017; Lazcano and Ackerman, 2018; Marler and Cruz, 2020). Studies of endangered cycad species have shown changes in vegetative growth and morphological traits are correlated with reproductive events (Clark and Clark, 1988) and population persistence in the wild (Raimondo and Donaldson, 2003). Conservation practitioners and researchers agree that understanding the factors that promote successful reproductive events is critical to the conservation of endangered plant species in general (Calonje *et al.,* 2011; Godefroid *et al.,* 2014), yet few studies have assessed the physiological changes that underlie reproductive activity and phenology in cycads. Creating such links will be critical to ongoing and future conservation efforts.

Resource allocation theory posits that individuals allocate limited resources to competing processes, such as growth, defense and reproduction, to maximize growth, survival and fitness (Reznick, 1985; Stearns, 1992; Obeso, 2002). Reproduction is thought to be an especially costly process in cycads because they generally undergo periods of thermogenesis to heat their strobili (Tang, 1987). Thermogenic periods function to volatilize compounds to attract pollinators (Seymour *et al.,* 2004; Suinyuy *et al.,* 2012), which then carry pollen from staminate strobili to ovulate strobili (Terry *et al.,* 2005). In cycads, staminate plants generally show more extreme characteristics related to thermogenesis of strobili compared with ovulate plants and can reach temperatures up to 10°C to 12°C above ambient temperature and contain overall greater concentrations of volatile compounds (Tang, 1987; Tang *et al.,* 1987; Roemer *et al.,* 2012).

Thermogenesis in cycads is sustained by converting stored carbon sources, such as starches into usable sugars (Tang, 1987). Moreover, recent work has shown that the timing of leaf production and reproductive events in *Cycas micronesica* may be particularly driven by depletion in non-structural carbohydrate (NSC) concentrations needed to construct vegetative and reproductive structures (Marler and Cruz, 2020). Despite the evidence for reproductive events being dependent on vegetative changes and carbon storage, the extent to which fertile individuals modulate photosynthetic carbon gain and which underlying traits best explain shifts in plant photosynthetic carbon gain remains unexplored in cycads.

Among the competing processes an organism must balance, the construction of reproductive structures has been shown to be particularly costly with respect to carbon and nitrogen resources. In addition to the conversion of stored starch to sugars (Tang, 1987; Marler and Cruz, 2020), studies have shown that plants can increase photosynthesis during reproduction (Gifford and Evans, 1981; Lehtila and Syrjanen, 1995; Laporte and Delph, 1996; Obeso, 2002; Raven and Griffiths, 2015). This increase in whole-plant photosynthetic output can be achieved in two, non-mutually exclusive ways, and has not been explored in cycads. For example, an individual cycad may acquire more carbon via greater photosynthetic capacity per leaf area, which could further be attributed to changes in, for example, stomatal conductance or the concentration of nutrients important for photosynthesis, such as nitrogen (Hikosaka, 2004; Wright *et al.,* 2004). Alternatively, plants could achieve greater whole-plant photosynthetic rates by simply increasing leaf number and area, as has been recorded in morphological differences between sterile and fertile individuals of *Zamia skinneri* (Clark and Clark, 1988). Understanding the mechanisms behind reproduction in cycads would be useful to contextualize the ecophysiology of gymnosperms and of other dioecious systems, as well as to better inform conservation practices (Gosetti *et al.,* 2022).

In some seed-free species that exhibit fertile-sterile leaf dimorphy, fertile leaves have been shown to be net carbon sinks with net carbon losses via respiration (Britton and Watkins Jr, 2016), suggesting that specialized reproductive tissues may require increased plant photosynthesis to compensate. Moreover, studies in other dioecious seed plant groups have measured physiological differences between sexes (Gehring and Monson, 1994), with ovulate plants presumed to incur higher reproductive costs and often exhibiting increased photosynthetic carbon gain capacity (Obeso *et al.,* 1998; Nicotra, 1999; Groen *et al.,* 2010). Given that dioecy is a highly specialized system of reproduction and previous work in cycads has shown that generating extra heat and compounds for thermogenesis is a costly process in terms of carbon expended, we hypothesize that staminate plants would exhibit increased rates of whole-plant photosynthesis during reproduction to help pay for the costs. We expected increased whole plant photosynthesis to be achieved by two primary mechanisms: 1) increased nitrogen content and 2) increased total leaf area in fertile vs sterile staminate plants.

## Methods

### Study species

*Zamia portoricensis* is a short-statured species of cycad endemic to the main island of Puerto Rico with known extant populations growing primarily on dry limestone soils (Supplementary Figure S1) and is pollinated by the beetle *Pharaxonotha portophylla* (Franz and Skelley, 2008). *Zamia portoricensis* is listed as endangered on the IUCN Red List with population extent and size declining in recent decades due to the destruction of native habitat, the intensification of the global plant trade and pollution from agriculture (Negrón-Ortiz *et al.,* 1996; Stevenson, 2010).

### Study location and sample selection

All individuals of *Z. portoricensis* included in this study were grown in an outdoor setting at Montgomery Botanical Center (MBC; Miami, FL, USA). We chose to focus on staminate individuals because of their generally greater levels of thermogenesis than ovulate plants across cycad species (Tang, 1987; Tang *et al.,* 1987; Roemer *et al.,* 2012). To conduct our measurements (see Supplementary Figure S2), we selected 12 staminate individuals of *Zamia portoricensis*, of which four were sterile (i.e. no reproductive organs) and eight were fertile (mature strobili present). We selected plants of roughly similar age; the average age of individuals in our study was 9.5 years with a standard deviation of 2.6 years. Age was determined by consulting the extensive cultivation database at the MBC. Within individuals, leaves were randomly selected for measurements among those that appeared healthy, fully expanded, and developmentally mature. Individuals were grown in a localized area in the garden in well-drained soil and under a common watering regime of supplemental irrigation and rain in partial sunlight. The climate is subtropical with an average annual temperature of 25°C and annual precipitation of ~ 157 cm. During the early summer months (late May to early June), when these data were collected, the average high temperature is ~ 30°C (US Climate Data, usclimatedata.com).

### Leaf morphology

To calculate stomatal density, from every individual, at least four leaf disks were cut from the leaflet directly adjacent and distal to those used in gas-exchange measurements using a 7-mm biopsy punch tool, and then were mounted in glycerol. Samples were examined with an Olympus BX-40 microscope (Olympus America Inc. Melville, NY, USA). Stomatal densities (D_s_) were measured using a ×40 objective using transmitted light.

We calculated the specific leaf area (SLA) of each individual by measuring the area of three fresh sampled leaflets within each individual and dividing that area by the dry mass of the leaflets after at least 72 hours in a drying oven at 65°C. We estimated the total leaf area of individual plants by measuring the area of one entire leaf from every fertile and sterile individual (n = 12) after all other measurements in the study had been taken and multiplying the species average leaf area by the number of leaves on each plant (including the sampled leaf). We combined leaf size measurements of sterile and fertile plants to calculate the average leaf area because there was no difference in the average leaf size between groups (data not shown).

Leaf number and average leaf area were used to scale additional parameters to the canopy level. Specifically, we scaled leaf-level photosynthetic rates to the canopy level by multiplying A_leaf_ with total canopy area of each individual. Canopy-level photosynthetic rates (A_canopy_) were converted from standard units (μmol CO_2_ m^−2^∙s^−1^) to mg CO_2_ m^−2^∙s^−1^ to avoid reporting large numbers in exponential notation in units of μmol CO_2_ m^−2^∙s^−1^. We also used the SLA and the estimated total leaf area of each individual to estimate the total leaf biomass for each individual in this study.

### Leaf nutrient content

Leaflet samples were analysed for nitrogen and carbon content (C) and carbon isotope discrimination (δ^13^C) following Krieg *et al.* (2017) using mass spectrometry facilities in the Department of Biology at Colgate University. Leaflet samples were oven dried at 65°C for 72 hours and ground into a powder using a Wiley Mill (Thomas Scientific Model 3383-L10). Ground samples were then weighed and rolled into tin capsules. Rolled samples were run through an elemental analyser, Costech Instruments Elemental Combustion System (Costech Analytical Technologies Inc. Valencia, CA, USA) in tandem with a Delta Plus Advantage Stable Isotope Mass Spectrometer (Thermo Fisher Scientific Inc. Waltham, MA, USA). Leaf isotope values are expressed in delta notation (‰) relative to the standard Pee Dee Belemnite and air, for C and N, respectively. Photosynthetic nitrogen-use efficiency (PNUE) was calculated by dividing leaf-level maximum photosynthetic rate by leaf nitrogen content.

### Gas exchange

We measured photosynthetic parameters on mature leaflets approximately 1/3 of the rachis down from the apex. We performed photosynthetic light-response curves over 15 different light intensities ranging from 0 to 1000 μmol∙m^−2^∙s^−1^ using a LI-6400 portable photosynthesis system (Li-Cor Biosciences Inc., Lincoln, NE, USA). These values were determined by running several test curves on the species to estimate light saturation point, thus measurements were limited to this range of light intensity because all individuals reached maximum values at a light level of ≤ 1000 μmol∙m^−2^∙s^−1^. All measurements were made with a flow rate of 400 μmol∙s^−1^, the CO_2_ mixer set to 400 ppm, and between 900 and 1300 hours. The LI-6400 was set to track ambient temperature and humidity inside the cuvette. Average leaf temperature inside the chamber (T_leaf_) during measurements was 31.8 (range, ± 2°C), with an average relative humidity of 65%. Light response curve data were fitted using a nonrectangular hyperbola model (de Lobo *et al.,* 2013) as implemented by the *fit_AQ_curve* function by N. Tomeo (https://github.com/Tomeopaste/AQ_curves). For each response curve, we recorded maximum photosynthetic rate per area (A_leaf_), light saturation point (LSP), light compensation point (LCP), light-use efficiency (ϕ), dark respiration (R_d_), as well as stomatal conductance (g_s_; at the time maximum A_leaf_ was achieved).

### Statistical analysis

We performed Shaprio-Wilk tests using the R package *rstatix* (Kassambara, 2021) and F-tests using the R function *var.test* (v3.6.2) on each variable in our data set to determine if it met the assumptions for parametric tests. All trait variables met the assumptions of a Student's *t*-test, thus, two-tailed Student's *t*-tests were used across our sterile vs. fertile comparisons. We also calculated Hedge's g to examine the size of the effect of each dependent variable using the R package *esvis* (Anderson, 2020). We chose Hedge's g over other effect size metrics (e.g. Cohen's d) because it has lower error rates when sample sizes are low (Hedges, 1981) (Table 1).

**Table 1:** Measurements of effect size for parameters tested

**Trait**	**Abbreviation**	**Units**	**Hedge’s g**
Leaf number	LN	# leaves	1.86*
Canopy photosynthetic rate	A_canopy_	mg CO_2_ m^−2^∙s^−1^	1.54*
Quantum yield	Φ	-	1.50*
Leaf nitrogen content	N	%	1.48*
Total leaf biomass		g	1.30*
Stomatal density	D_s_	# stomata mm^−2^	0.88
Leaf photosynthetic rate	A_area_	μmol∙m^−2^∙s^−1^	−0.63
δ13C	δ13C	‰	0.57
Stomatal conductance	g_s_	μmol∙m^−2^∙s^−1^	−0.46
Leaf respiration rate	R_d_	μmol∙m^−2^∙s^−1^	0.29
Specific leaf area	SLA	cm^2^∙g^−1^	0.028
Light compensation point	LCP	μmol∙m^−1^∙s^−1^	0.46

The parameters are sorted by effect size, with greater Hedge’s g values representing greater effects of fertility for a given trait, regardless of direction (+/−): small effect ≥0.2, medium effect ≥0.5, and a large effect ≥0.8, following Cohen (1977) and Hedges and Olkin (1985). Asterisks (*) indicate statistically significant effects

We also tested for multivariate differences between sterile and fertile individuals. Before performing multivariate analyses, we checked for multicollinearity between dependent variables by examining pairwise correlations using the *rcorr* function from *Hmisc* package (Harrell and JrDupont, 2021). We removed a variable when there was a pairwise correlation (R^2^) above 0.70. The variables with correlations above this threshold and thus were removed, were leaf number and PNUE. We chose to remove leaf number because it was highly correlated with canopy-level photosynthesis and because we are not aware of any study that has examined canopy-level photosynthesis in sterile and fertile cycads, in contrast to examining leaf number (Tang, 1990). We chose to remove PNUE because it was highly correlated with the two input variables to its formulation, leaf-level photosynthetic rate and leaf nitrogen content, but leaf nitrogen content and leaf-level photosynthesis were not correlated above 0.7 (Figure 5), thus, removing only PNUE retained more data. After removing highly correlated variables, we performed a Hotelling's multivariate t-test using the *Hotelling* package (Curran, 2018). The complete set of variables included in the analyses can be found in Supplementary Table 1. To graph and visualize our results, we used the R packages *ggplot2* (Wickham *et al.,* 2016). We visualized these same variables in multivariate space using a principal component analysis (PCA) with the *stats* R package (R Core Team, 2022, v4.2.0).

## Results

### Leaf morphology

**Figure 1:**
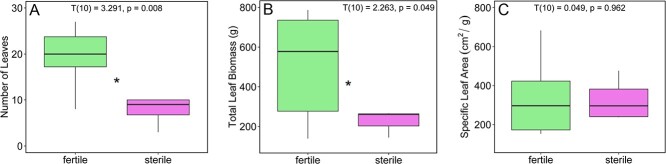
(A) Number of leaves, (B) Leaf biomass, (C) Specific leaf area.

**Figure 2:**
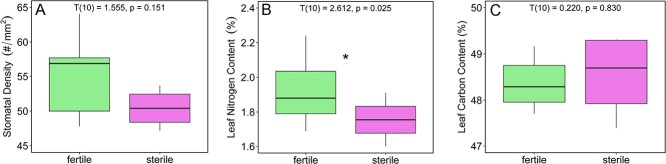
(A) Stomatal density, (B) Leaf nitrogen content, (C) Leaf carbon content.

Fertile plants generally had more leaves (T(10) = 3.291, p = 0.008, g = 1.86; Fig. 1A, B), larger total leaf area (T(10) = 3.745, p = 0.004, g = 2.15) and greater leaf biomass (T(10) = 2.263, p = 0.049, g = 1.3; Fig. 1B) compared to sterile plants. However, there was no difference in SLA measurements between the two groups (T(10) = 0.049, p = 0.962, g = 0.028; Fig. 1C). There were no significant differences in the stomatal density between sterile and fertile individuals (T(10) = 1.555, p = 0.151, g = 0.879; Fig. 2A).

### Leaf nutrient content

Fertile plants had greater nitrogen content than sterile plants (T(10) = 2.612, p = 0.026, g = 1.48 (Fig. 2B), although carbon content was not different between groups (T(10) = 0.221, p = 0.83, g = 0.125) (Fig. 2C). Levels of carbon isotope discrimination (δ^13^C) (T(10) = 1.003, p = 0.339, g = 0.567) and nitrogen isotope discrimination (δ^15^N) were not different between sterile and fertile individuals (T(10) = 0.816, p = 0.433, g = 0.461).

### Gas exchange

**Figure 3:**
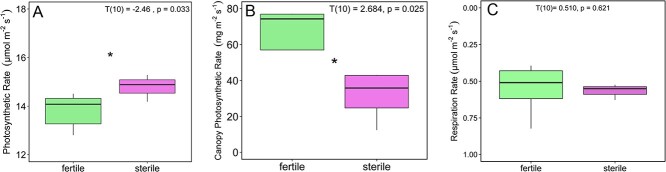
(A) Leaf photosynthesis, (B) Canopy photosynthesis, (C) Dark respiration.

**Figure 4:**
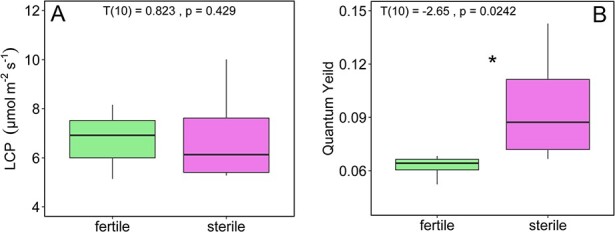
(A) Quantum yield, (B) LCPs.

**Figure 5:**
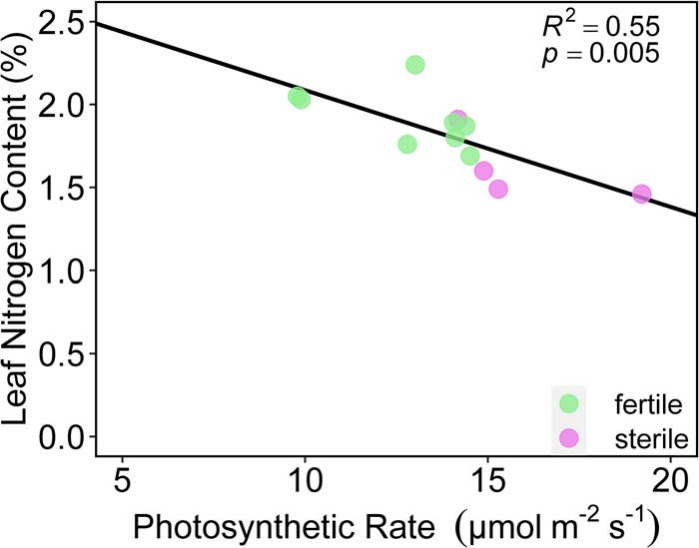
Leaf-level photosynthesis vs leaf nitrogen content.

Although sterile plants achieved higher leaf photosynthetic rates (A_leaf_) than fertile plants (T(10) = −2.46, p = 0.033, g = −1.39; Fig. 3A), canopy-level photosynthesis was significantly greater in fertile plants than in sterile plants (T(10) = 2.684, p = 0.025, g = 1.54; Fig. 3B). Respiration rates (R_d_) were not different between fertile and sterile individuals (T(10) = −0.743, p = 0.474, g = 0.420; Fig. 5A), nor were PNUEs (T(10) = −1.839, p = 0.099, g = −1.05), nor were maximum stomatal conductances (g_s_) (T(10) = −0.821, p = 0.431, g = −0.464). Additionally, the two groups did not vary significantly with regard to their instantaneous water use efficiency (calculated as A_leaf_/E) (T(10) = −0.600, p = 0.556, g = −0.515). Sterile individuals achieved higher light-use efficiency compared with fertile individuals (T(10) = −2.65, p = 0.0242, g = −1.50 Fig. 4A) but we detected no difference in LCP (T(10) = 0.823, p = 0.429, g = 0.465; Fig. 4B). There was a negative correlation between nitrogen content and photosynthesis rates across all individuals (R^2^ = 0.33, p = 0.028; Fig. 5), but no correlation in fertile plants only (R^2^ = 0.28; p = 0.17; Fig. 5) or sterile plants only (R^2^ = 0.44; p = 0.33; Fig. 5).

### Multivariate comparisons

Hotelling’s multivariate *t*-test detected significant differences between fertile and sterile individuals (T = −0.591, p = 0.026, with 10 000 permutations). We included 12 dependent variables in our multivariate test between sterile and fertile individuals: leaf-level photosynthetic rate (A_leaf_), stomatal conductance (g_s_), respiration rate (R_d_), light-use efficiency (ϕ), LCP, leaf nitrogen and carbon content (N% and C%), leaf nitrogen composition (δ^15^N), specific leaf area (SLA), stomatal density (D_s_), total vegetative biomass (Biomass) and canopy level photosynthetic carbon gain (A_canopy_).

## Discussion

Numerous studies have shown that reproduction can be energetically costly. In some cases, such costs can impose constraints on allocation patterns and impact physiological function across diverse plant groups (Givnish, 1980; Reznick, 1985; Campbell, 2000). However, the link between energy acquisition, canopy size (i.e. total plant leaf area) and reproductive status in cycads is poorly understood (Newell, 1985; Clark and Clark, 1988; Krieg *et al.,* 2017; Marler and Cruz, 2020; Gosetti *et al.,* 2022), and little is known about the changes in energy budget through reproductive phases in either sex, particularly with respect to photosynthetic physiology. Our results build on previous research that shows increased leaf production and leaf number coincides with reproductive periods in some cycads (Newell, 1985; Clark and Clark, 1988; Tang, 1990). We found that this increase in total leaf area in staminate individuals of *Z. portoricensis* results in a significantly higher canopy-level photosynthetic rate despite a lower photosynthetic rate per area (Fig. 1A, Fig. 3A, B). This increase in whole-plant carbon gain may have significant impacts on an individual's carbon budget and ability to accumulate NSCs. Studies in other plant groups have shown that the demand for carbon is relatively high during reproductive events (Obeso, 1997; Sunmonu *et al.,* 2013; Yu *et al.,* 2013) and staminate plants of *Z. portoricensis* may acquire much of their carbon budget during this period. However, an increased demand for carbon during fertile phases should therefore also coincide with increased carbon expenditure. Marler and Cruz (2020) found that NSC concentrations decreased during periods of reproduction in staminate individuals of Cycas *micronesica*, indicating that mobilization and conversion of NSCs through respiration may be critical to fuel thermogenesis and/or construction of reproductive structures.

Decades of photosynthesis research have found that increased photosynthetic capacities generally require greater respiration rates (Benson and Calvin, 1950; Amthor, 2000; von Caemmerer *et al.,* 2009; Poorter *et al.,* 2019). If fertile individuals of *Z. portoricensis* are mobilizing or metabolizing NSCs similar to *Cycas micronesica* (Marler and Cruz, 2020) then we would also expect fertile individuals to have relatively high respiration rates. Indeed, despite having a lower maximum leaf-level photosynthetic rates compared to sterile individuals, fertile individuals had respiration rates that were just as high as sterile leaves (Fig. 3A, C). Given that respiration is the primary mechanism by which leaves convert photosynthetically fixed carbon into the energy required for growth, maintenance and reproduction, this suggests that fertile plants may be experiencing a greater demand for carbon than sterile plants. To date, no study has tracked the accumulation, expenditure and allocation of carbon in any cycad species with respect to leaf production and reproduction. These processes deserve more attention.

**Figure 6:**
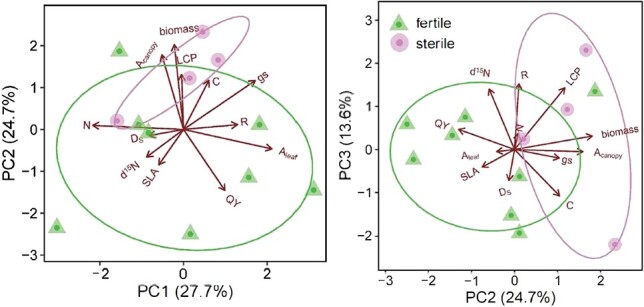
Principal component analysis.

Fertile individuals achieve greater rates of photosynthetic carbon gain by nearly doubling the number of leaves (Fig. 1A), which is also a costly investment that would require significant inputs of carbon. Many studies have shown the carbon costs of constructing leaves can vary but limited data suggest that the construction cost of leaves in cycads is generally higher than many other vascular plants (Meng *et al.,* 2021) because of their generally very long lifespans. The construction cost of leaves on fertile and sterile individuals of *Z. portoricensis* are likely similar, given that they had similar specific leaf areas and leaf carbon content (Fig. 1C, Fig. 2C). However, sterile individuals had leaf-level carbon fixation rates nearly 30% higher than fertile leaves and higher photosynthetic carbon gain per investment in leaf biomass (Fig. 1A, Fig. 3A, B) which may be critical to reaching the carbon and NSC threshold required to produce reproductive structures and leaves during reproductive phases. However, Zamia species can have large underground storage organs (Whitelock, 2002) and studies in other plant groups, like ferns, have shown that leaf production is achieved by expending carbon that was fixed and stored in prior years (Britton and Watkins Jr, 2016). The time course and translocation of carbohydrates and other nutrients is not well understood in cycads (Gosetti *et al.,* 2022).

Although fertile plants had higher canopy level photosynthesis (Fig. 3A), which they achieved through more total leaf area (Fig. 1A), sterile plants had higher maximum leaf-level photosynthetic rates (A_leaf_) and light-use efficiency (ϕ) than fertile plants (Fig. 3A, Fig. 4A). By taking a multi-trait approach, we were able to rule out the potential impact of several traits on photosynthetic capacity. For example, many studies have documented the positive impact of nitrogen content, stomatal density and/or stomatal conductance on leaf gas exchange (Hirose *et al.,* 1997; Franks and Beerling, 2009; Buckley and Mott, 2013; Reich, 2014). However, an intriguing pattern that emerged from our data is the greater photosynthetic rate of sterile individuals (Fig. 3A), despite having similar stomatal density and conductance (Fig. 2A, Supplementary Figure S3) and lower nitrogen content (Fig. 2B) relative to fertile individuals. In addition, when we plotted leaf nitrogen content against photosynthetic rates, there was a negative correlation at the leaf level (Fig. 5). Interestingly, it seems that the difference in nitrogen content between sterile and fertile staminate *Z. portoricensis* does not increase photosynthetic rates. It is possible that higher whole-plant photosynthetic rates observed in fertile individuals could lead to more C available for the N-fixing symbionts that have been widely observed in cycads (Whitelock, 2002), which could then lead to greater leaf nitrogen in fertile plants. Ultimately, the causes of the difference in nitrogen levels in these sterile and fertile staminate plants are unclear, and deserve more research.

Understanding better the reproduction of cycads is a priority for conservation organizations and can help inform efforts to provide endangered cycads with the specific resources and conditions to better facilitate reproduction (Calonje *et al.,* 2011; Godefroid *et al.,* 2014). For example, we showed that reproductive phases coincide with changes in whole-plant morphology and photosynthetic physiology in the endangered cycad species, *Zamia portoricensis* (Table 1, Fig. 6). In particular, *Z. portoricensis* showed temporal variation in physiological adaptations associated with light-limited environments, e.g. increased light-use efficiency and capacity of leaves during sterile phases. We speculate that this shift in photosynthetic physiology could have important impacts on the ecology and reproduction of this species in light-limited environments. Further research is required to quantify the timing of acquisition, translocation and allocation of carbon that is used for reproduction and leaf development so that conservation practitioners could best promote leaf growth and reproduction in this species. For example, future research could make similar measurements to those reported here over longer timescales to account for physiological differences between leaves of different developmental ages (Krieg *et al.,* 2023), while individual plants alternate between fertile and sterile reproductive states over time.

## Supplementary Material

Web_Material_coad020

## Data Availability

Our data are included as a Supplementary Data File and available online in the Zenodo data repository (10.5281/zenodo.7698749).
