# Supplementary material for: Reproductive phases coincide with changes in morphology and photosynthetic physiology in an endangered cycad species
Source: Conserv Physiol. 2023 Apr 25;11(1):coad020. doi: 10.1093/conphys/coad020 (PMC10132820; doi:10.1093/conphys/coad020)
Supplement: Web_Material_coad020 [file web_material_coad020.zip › Supplementary_Information_R2_clean.pdf]

Supplementary Information

Reproductive phases coincide with changes in morphology and photosynthetic physiology in an endangered cycad species

Christopher P. Krieg[1]*, Sophia Gosetti[1,2], James E. Watkins Jr.[3], M. Patrick Griffith[4], Katherine A. McCulloh[1]

1 Department of Botany, University of Wisconsin, Madison, WI 53706, USA
2 Glacial Lakes Conservancy, Sheboygan, WI 53081, USA
3 Department of Biology, Colgate University, Hamilton, NY 13346, USA
4 Montgomery Botanical Center, Coral Gables, FL 33156, USA
*correspondence: christopher.p.krieg@gmail.com

Supplementary Tables and Figures:

- Supplementary Figure 1: Distribution of *Z. portoricensis* in southern Puerto Rico
- Supplementary Figure 2: Methods and Sampling Infographic
- Supplementary Figure 3: Stomatal conductance
- Supplementary Figure 4: Leaf carbon isotope discrimination
- Supplementary Data File: summary statistics of the dataset in .csv format

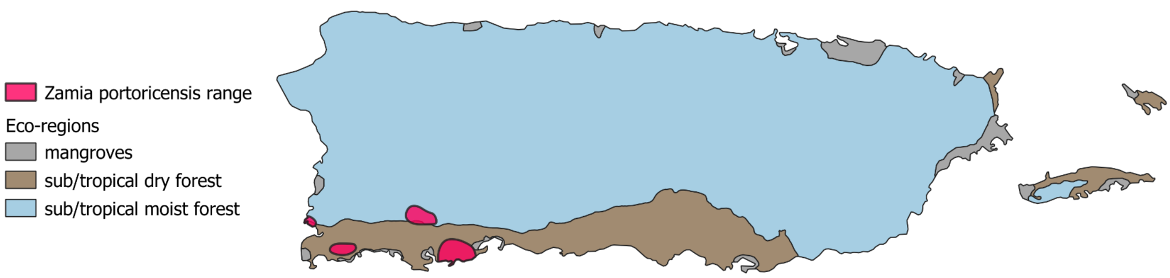

Figure S1. Geographic and ecological distribution of *Zamia portoricensis* in Puerto Rico. Areas colored fuchsia represent geographic areas with populations of *Z. portoricensis*. The landscape of Puerto Rico is colored by ecoregion. Species location data were gathered primarily from the literature to supplement limited georeferenced point location data from large databases (e.g. GBIF.org).

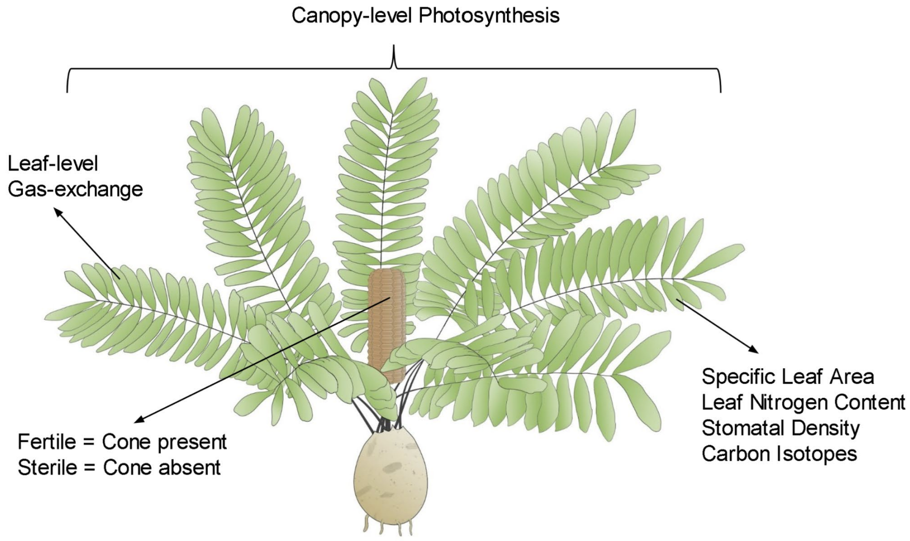

Figure S2. Graphical presentation of a generic Zamia species (i.e., this is not a reference illustration of Zamia *portoricensis*) with indications of general sampling location and methodological details of select trait measurements. Cycad illustration by Jerald Pinson, Ph.D. (www.jeraldpinson.com). See Methods section of Main Text for more information.

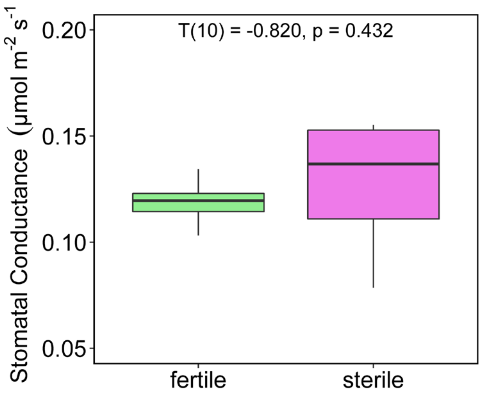

Figure S3. Boxplot of stomatal conductance in sterile (violet) and fertile (lime green) individuals of *Z. portoricensis*. No differences were found (T(10)=-0.820, p = 0.431).

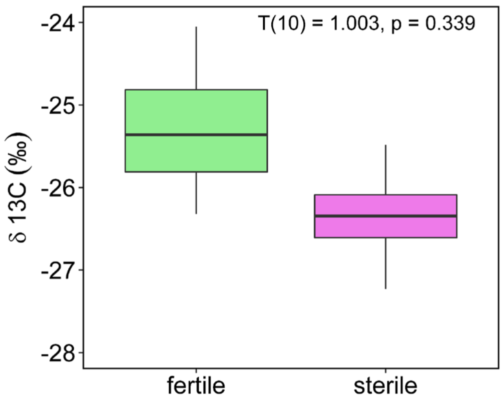

Figure S4. Boxplot of leaf carbon isotope discrimination (‰) in sterile (violet) and fertile (lime green) individuals of *Z. portoricensis*. No differences were found in leaf carbon isotopes (T(10)=1.00, p = 0.34).

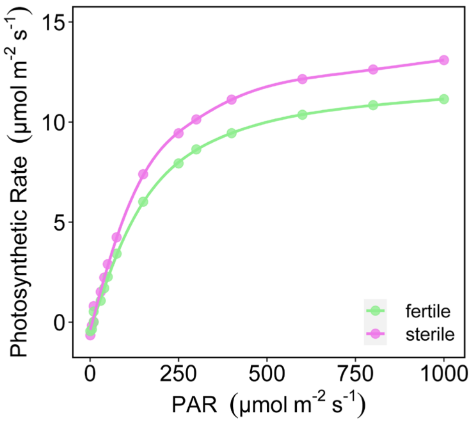

Figure S5. Mean photosynthetic rates per area as a function of light intensity in sterile (violet) and fertile (lime green) individuals of *Z. portoricensis*. See results section in main text for comparisons of derived parameters.

Supplementary Information

Supplementary Data File. descriptive statistics and data used in this study.
